# Disease Variant Landscape of a Large Multiethnic Population of Moyamoya Patients by Exome Sequencing

**DOI:** 10.1534/g3.115.020321

**Published:** 2015-10-29

**Authors:** Lorelei D. Shoemaker, Michael J. Clark, Anil Patwardhan, Gemma Chandratillake, Sarah Garcia, Rong Chen, Alexander A. Morgan, Nan Leng, Scott Kirk, Richard Chen, Douglas J. Cook, Michael Snyder, Gary K. Steinberg

**Affiliations:** * Department of Neurosurgery and Stanford Stroke Center, Stanford University, Stanford, California 94305; † Personalis Inc., Menlo Park, California 94025; ‡ Stanford Medical School, Stanford University, Stanford, California 94305; § Centre for Neuroscience Studies and the Department of Surgery, Queen's University, Kingston, Ontario, K7L 3N6; ** Department of Genetics, Stanford University, Stanford, California 94305

**Keywords:** cerebrovascular disease, hemorrhage, RNF213, ZXDC, OBSCN

## Abstract

Moyamoya disease (MMD) is a rare disorder characterized by cerebrovascular occlusion and development of hemorrhage-prone collateral vessels. Approximately 10–12% of cases are familial, with a presumed low penetrance autosomal dominant pattern of inheritance. Diagnosis commonly occurs only after clinical presentation. The recent identification of the *RNF213* founder mutation (p.R4810K) in the Asian population has made a significant contribution, but the etiology of this disease remains unclear. To further develop the variant landscape of MMD, we performed high-depth whole exome sequencing of 125 unrelated, predominantly nonfamilial, ethnically diverse MMD patients in parallel with 125 internally sequenced, matched controls using the same exome and analysis platform. Three subpopulations were established: Asian, Caucasian, and non-*RNF213* founder mutation cases. We provided additional support for the previously observed *RNF213* founder mutation (p.R4810K) in Asian cases (*P* = 6.01×10^−5^) that was enriched among East Asians compared to Southeast Asian and Pacific Islander cases (*P* = 9.52×10^−4^) and was absent in all Caucasian cases. The most enriched variant in Caucasian (*P* = 7.93×10^−4^) and non-*RNF213* founder mutation (*P* = 1.51×10^−3^) cases was *ZXDC* (p.P562L), a gene involved in MHC Class II activation. Collapsing variant methodology ranked *OBSCN*, a gene involved in myofibrillogenesis, as most enriched in Caucasian (*P* = 1.07×10^−4^) and non-*RNF213* founder mutation cases (*P* = 5.31×10^−5^). These findings further support the East Asian origins of the *RNF213* (p.R4810K) variant and more fully describe the genetic landscape of multiethnic MMD, revealing novel, alternative candidate variants and genes that may be important in MMD etiology and diagnosis.

Moyamoya disease (MMD) is a rare cerebrovascular disease characterized by progressive bilateral and occasionally unilateral stenosis and occlusion of the supraclinoidal internal carotid artery (ICA), with frequent involvement of the middle cerebral artery (MCA) and anterior cerebral artery (ACA), and by the development of fragile, abnormal collateral vessels. Patients are at risk for hemorrhagic or ischemic stroke, seizures, and developmental delays. Pathologically, the affected vasculature exhibits smooth muscle cell (SMC) hyperplasia and a disrupted or duplicated elastic lamina (Yamashita *et al.* 1983). Patients with disorders such as Down syndrome, sickle cell disease, neurofibromatosis type 1 (NF-1), and microcephalic osteodysplastic primordial dwarfism type II (MOPD II) have a higher incidence of MM angiopathy, collectively termed MM syndrome (MMS) (Scott and Smith 2009). MMD was first described in Japan in the 1950s and was once thought to be prevalent only in the Japanese population, although it has since been identified in many ethnic groups, including European, East Asian, Southeast Asian, Pacific Islander, Korean, Caucasian, African-American, and Hispanic (Scott and Smith 2009; Suzuki and Takaku 1969). In Japan, MMD has an incidence of 1.13/100,000 and a prevalence of 5.22/100,000 (Hayashi *et al.* 2013). The incidence of MMD in the western United States was only 0.086/100,000 in a 2005 survey, but more recent data suggest an incidence of 0.57/100,000 in the United States nationwide (Uchino *et al.* 2005; Starke *et al.* 2012). Approximately 10–12% of MMD cases are familial, with a presumed low penetrance autosomal dominant pattern of inheritance (Scott and Smith 2009), and a number of MMD-associated loci have been reported, including 3p24.2-p26, 6q25, 8q23, 12p12, and 17q25.3 (Ikeda *et al.* 1999; Inoue *et al.* 2000; Sakurai *et al.* 2004; Mineharu *et al.* 2008; Yamauchi *et al.* 2000). Mutations have been found in specific genes, including an Xq28 deletion that affects both the *BRCC3* and *MTCP1/MTCP1NB* genes in a particular MMS (Miskinyte *et al.* 2011), and variants of *ACTA2*, which were present in a mixed, non-Asian population but not in a Japanese population (Roder *et al.* 2011; Guo *et al.* 2009; Shimojima and Yamamoto 2009). *RNF213* (Gene ID: 57674) was identified as the first MMD-associated gene in an Asian MMD population (Kamada *et al.* 2011; Liu *et al.* 2011; Wu *et al.* 2012) and, although its exact function is not known, morpholino-based knockdown in zebrafish resulted in defects in angiogenesis (Liu *et al.* 2011), and while mouse knockout models showed no cerebrovascular phenotype, recent evidence suggests increased angiogenesis in a hind-limb ischemia model (Kobayashi *et al.* 2013; Ito *et al.* 2015).

Despite these advances, MMD continues to be challenging to diagnose and its etiology is still not well understood [reviewed in Guey *et al.* (2015), Houkin *et al.* (2012), and Fujimura *et al.* (2014)]. To further identify the genetic components of MMD, we internally and in parallel sequenced the exomes of 125 unrelated, predominantly nonfamilial, ethnically diverse patients and 125 matched normal controls from the Personalis Control Library. Due to the rarity of MMD and the diversity of patients in our cohort, we sought to identify additional rare, high-penetrance founder mutations (similar to the known RNF213 p.R4810K variant) in the ethnic groups. We also sought to discover additional associated genes in a case/control study by focusing on clustering of rare, low-penetrance variants. We also fully characterized mutations in RNF213 across the diverse set of cases to test whether additional founder mutations exist in other ethnic populations. Future studies will further elucidate the roles of these variants in MMD across ethnicities.

## Materials and Methods

### Patient demographics and clinical data

Samples were obtained under consent and with approval from Stanford’s Institutional Review Board from MMD patients undergoing bypass surgery in the Department of Neurosurgery at Stanford. MMD was diagnosed angiographically based on the presence of typical bilateral and occasionally unilateral stenosis/occlusion of the ICA, ACA, and/or MCA, together with the presence of collateral lenticulostriate perforating arteries. Of the 125 MMD patients, approximately 35% of the MMD patients were diagnosed with hypertension and/or hyperlipidemia. No MMD patients had a recorded history of cranial irradiation. The cases were predominantly sporadic, with 13 unrelated patients with familial MMD. A few (11) were also diagnosed with MMD-associated conditions such as Graves disease and Down syndrome. Self-reported case ethnicities were further confirmed by principal components analysis (PCA)-based clustering. The Personalis Control Library comprised DNA obtained through the 1000 Genomes project and was sequenced and analyzed internally with the MMD samples.

### Study sample selection and demographics

The 125 ethnically diverse, unrelated MMD patients were matched based on sex and a broad ethnic category to 125 controls obtained from the Personalis Controls Library constructed from DNA obtained from the 1000 Genomes Project (The 1000 Genomes Project Consortium 2012). The three subsets comprised 35 MMD cases of East Asian/Southeast Asian/Pacific Islander ethnicity, 68 MMD cases of Caucasian/South Asian ethnicity, and 91 MMD multiethnic cases without the *RNF213* (p.R4810K) founder mutation (Table 1).

**Table 1:** Summary of MMD and control groups demographics

	East Asian/Southeast Asian/Pacific Islander		Caucasians		Subjects without *RNF213* Founder Mutation	
	**Case**	**Control**	**Case**	**Control**	**Case**	**Control**
**Total**	35	35	68	68	91	91
**Age**	38.5 (22.5–49.8)*^a^*	*^b^*	37 (23.5–48)	*^b^*	39 (24–50)	*^b^*
						
**Age at disease onset**	25 (6–41.3)	NA	24 (4.8–37)	NA	24 (5–38)	NA
**Female**	23 (66%)	23 (66%)	48 (71%)	48 (71%)	63 (69%)	63 (69%)

a Median (interquartile range).

b Age-related information not available from Personalis Control Group samples.

### Genomic library construction

Genomic DNA was extracted using the Gentra Puregene kit (Qiagen, Valencia, CA). Libraries were prepared from approximately 3 µg of high-quality genomic DNA (50–200 ng/μl) using Illumina TruSeq Genomic DNA High Throughput Sample Prep Kits (Illumina, San Diego, CA), and exome enrichment (targeting 62 Mb) was accomplished using the TruSeq Exome Target Enrichment kit (Illumina, San Diego, CA); all were performed according to the manufacturer’s protocols. Target enrichment validation was confirmed by determining the concentration of the library by PicoGreen-based quantitation. Library yields ranged from 100–1000 ng of DNA, a portion of which was run on the Bioanalyzer HS DNA chip (Agilent, Santa Clara, CA), with an average size of 300–550 nt for DNA fragments.

### Exome sequencing

Sequencing was performed using Illumina Hiseq2000 or HiSeq2500 sequencers with single lane, paired-end 2×100 bp reads. DNA fragments were generated and amplified using Clonal Single Molecule Array technology (Illumina, San Diego, CA). The sequences were determined using the Clonal Single Molecule Array and Sequencing-by-Synthesis using Illumina’s instrumentation and Reversible Terminator Chemistry. Each sequencing lane interrogated the DNA sequences of a pool of three individual sample libraries each carrying a unique index. Sequencing reads of at least 2×100 bp in length for a total of approximately 8 Gb of sequence data were generated for each sample. To improve the accuracy of variant calling, control DNA from the Personalis Control Library was sequenced internally on the same exome platform utilizing the same sequencing technology and analysis pipeline as the MMD cases.

### Alignment and variant calling

For all platforms, raw sequence data were in FASTQ format and were analyzed with standard Phred-scale quality scores. Gapped alignment to the hg19 genome was performed using the Burrows-Wheeler Aligner combined with Picard and the Genome Analysis Toolkit (GATK) base quality score recalibration to perform sequence alignment and base quality scoring. GATK’s Unified Genotyper module provided the core set of SNV and InDel calls and quality metrics using both GATK’s variant quality score recalibration (VQSR) (for SNVs) and hard-filtering (for InDels), per GATK best practices documentation. The mean coverage depth (∼100×) was calculated from the base-resolved coverage depth integrated over the exon length, considering only aligned bases with high-quality mapping (Q ≥20) and base-quality (Q ≥20) scores. The variants were then annotated with the Personalis Annotation Engine, which supplied population frequencies, genetic region information, effect on genes, protein impact, known disease association, protein–protein interactions, and additional genetic features to the variants.

### Case-control analysis

The control and case raw sequencing data were aligned and variants were called simultaneously. Analysis was performed independently in each of the three matched case-control studies to investigate variant and gene associations with MMD. Variants were removed from analysis if the call quality failed our internal quality control (QC) criteria or if more than 30% of the data were missing across samples. Remaining variants were assessed for association with disease status using Fisher’s exact test. Effect size was summarized as the OR calculated from the conditional maximum likelihood estimate of a 2×2 contingency table containing alternative and reference allele counts in cases and controls assuming an additive model. Significance testing of the null hypothesis of conditional independence (OR = 1) used a two-tailed test with *P* values obtained directly from a hypergeometric distribution. In instances where zero cell counts were encountered, either zero or infinite values for the OR were obtained.

MMD is a rare, genetically heterogeneous monogenic disease with few disease-causing variants, so we applied additional variant inclusion criteria to reduce false discoveries when reporting MMD-associated variants and genes. Variants were retained if they: (1) were protein-coding SNPs; (2) had a minor allele frequency (MAF) <5% based on ethnic-specific 1000 Genome frequency information; and (3) were enriched in MMD cases. By these criteria, a known MMD allele in the Asian population would be identifiable in previously described studies (*RNF213*, p.R4810K by the 1000 Genomes has a MAF = 0.001 in all populations and a MAF = 0.002 in Asian population) (Kamada *et al.* 2011; Liu *et al.* 2011, 2012; Wu *et al.* 2012; The 1000 Genomes Project Consortium 2012).

We collapsed the variant-level associations based on gene membership using the Combined Multivariate and Collapsing (CMC) method (Li and Leal 2008). Variants were binned into groups based on their respective gene associations. A multivariate test, Hotelling T2, was performed on the counts within all bins to determine differences among the cases and controls and asymptotic *P* values were calculated based on the F-distribution. The method of Storey (2002) was used to calculate false discovery rate (FDR)-adjusted *P* values (*i.e.*, q-values).

Protein sequence data were obtained from the UniProtKB (www.expasy.ch) using only reviewed entries.

## Results

### Identification of genetic variants in Asian, Caucasian, and non-*RNF213* founder mutation MMD cases

To determine the genetic variants associated with MMD, we performed high-depth whole exome sequencing on 125 ethnically diverse, unrelated, and predominantly nonfamilial MMD patients and 125 sex- and ethnicity-matched controls from the 1000 Genomes Project (The 1000 Genomes Project Consortium 2012). Patient samples were taken from a set of archival tissues, and controls were randomly selected using 1:1 case:control matching based on sex and ethnicity. Samples were divided into two ethnically matched case-control studies: an East Asian, Southeast Asian, and Pacific Islander study (*n=*70) and a Caucasian study (*n=*136). A third pan-ethnic study with cases and controls selected on the lack of the *RNF213* (p.R4810K) founder mutation was also performed (*n*=182). The case and control demographics are outlined in Table 1 and the subethnicities are outlined in Supporting Information, Table S1. MMD cases ranged from 22 to 50 years of age. Age-related data for the control cases were not available but are assumed to cover a wide range. MMD is known to affect more females than males (Khan *et al.* 2012), and as a result our study contained approximately twice as many females as males (Table 1).

**Figure 1:**
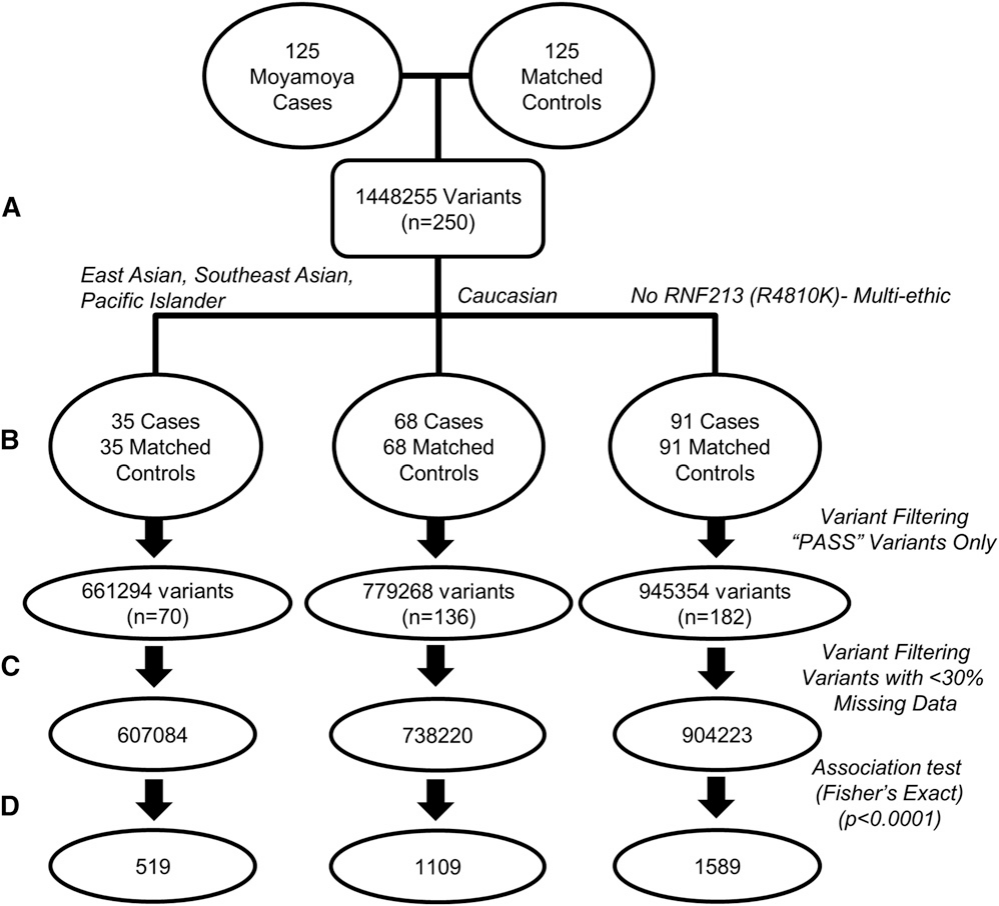
Case-control workflow for variant discovery. There were 125 MMD cases individually matched based on ethnicity and sex to 125 subject samples drawn from the Personalis Controls Library. Samples were analyzed in a stepwise process of exome sequencing, variant alignment and detection, and variant annotation to obtain 1,448,255 variants across 250 samples (A). This sample set was subdivided to construct three case-control datasets based on ethnicity (East Asian/Southeast Asian/Pacific Islander; Caucasian) and on the lack of the *RNF213* founder variant (B). In each of these case-control datasets, variants were filtered to include only those with adequate call quality and excluded those with >30% missing data across samples (C). These variant sets were used to create a list of enriched variants (*P* < 0.0001) for each case-control study assuming an additive model (D).

Using an optimized, stepwise process of exome sequencing, read alignment, variant detection, and annotation, a total of 1,448,255 variants were identified in these 250 samples (Figure 1A). The variants for each case-control study were extracted, creating three subsets (Figure 1B). A series of filtering steps removed variants that failed to pass filters and those with greater than 30% of samples missing data (Figure 1C). A genetic similarity test confirmed that self-reported ethnicities generally clustered according to genetic similarity (Figure S1). Two pairs of self-declared Caucasian MMD samples were sibling pairs (Figure S1, A and B), and only one member of each sibling pair was used for downstream case/control analysis. Two self-declared Hawaiian cases and one Admixed American case clustered with the Korean and Japanese cases, and all three were determined to be of East Asian descent by genetic similarity (Figure S1, C and D). An association test was then performed, identifying 519 variants in the East Asian/Southeast Asian/Pacific Islander dataset, 1109 variants in the Caucasian dataset, and 1589 variants in the non-*RNF213* founder mutation dataset, all significant at *P* ≤ 0.0001 (Figure 1D).

### The *RNF213* (p.R4810K) founder mutation was present only in East Asian MMD cases

The *RNF213* (p.R4810K; rs112735431) founder mutation was the most enriched (*P* = 6.01×10^−5^) variant identified in East Asian MMD (Table 2) and was present in 14 (56%) of the 25 cases, which included those individuals determined to be of East Asian descent by genetic clustering (Table 3), confirming previously published work (Liu *et al.* 2011, 2012; Kamada *et al.* 2011; Cecchi *et al.* 2014; Liu *et al.* 2013). The *RNF213* (p.R4810K) founder mutation was identified only in specific ethnic groups, specifically in self-declared Korean (73%), Japanese (60%), and Chinese (20%) MMD cases (Table 3). The mutation was also observed in one self-declared Hawaiian (Figure S1C) and one self-declared Admixed American (Figure S1D) case, both determined to be of East Asian descent based on genetic similarity. Among Asian cases, we did not observe the *RNF213* (p.R4810K) founder mutation in any individuals outside the East Asian ethnic subpopulation, including those cases in the Southeast Asian and Pacific Islander subgroup (Filipino, Vietnamese, Cambodian, Laotian, Malaysian, and Hawaiians, *n* = 13), as illustrated in Table 3. Notably, the *RNF213* founder mutation was completely absent from Caucasian MMD cases (*n=*74) as well as in the smaller sets of African American (*n=*5), South Asian (*n=*3), and Admixed/Native American (*n=*6) MMD cases (Table 3). In support of the low penetrance dominant mode of inheritance in MMD, the *RNF213* founder mutation was present infrequently in controls (9 of 125).

**Table 2:** Most enriched nonsynonymous variants in MMD cases within the East Asian/Southeast Asian/Pacific Islander, the Caucasian, and the non-*RNF213* founder variant subpopulations

								Cases			Controls				
dbSNP	Chromosome Position	Gene Symbol	Ref/Alt Allele	Amino Acid Change	Freq Overall	Freq Asian	Freq European	AA	Aa	aa	AA	Aa	aa	OR (95% C.I.)	*P* value
**East Asian/Southeast Asian/Pacific Islander cases**															
rs112735431	Chr17:78358945	*RNF213*	G/A	p.R4810K	0.0009	0.0035	(NA)	1	12	22	0	0	35	Inf	6.01×10^−5^
rs140134109	Chr19:7565823	*C19orf45*	A/G	p.M39V	0.010	0.040	(NA)	0	9	26	0	0	35	Inf	2.98×10^−3^
rs146586179	Chr7:148975615	*ZNF783*	G/A	p.A267T	0.010	0.030	(NA)	1	6	28	0	0	35	Inf	6.34×10^−3^
rs2241012	Chr17:36868139	*MLLT6*	G/A	p.A198T	0.010	0.020	(NA)	1	6	28	0	0	35	Inf	6.34×10^−3^
rs12718465	Chr11:116707736	*APOA1*	C/T	p.A61T	0.010	0.040	(NA)	0	7	28	0	0	35	Inf	1.33×10^−2^
**Caucasian cases**															
rs16837497	Chr3:126180820	*ZXDC*	G/A	p.P562L	0.040	(NA)	0.040	1	9	58	0	0	68	Inf	7.93×10^−4^
rs143744326	Chr1:152128212	*RPTN*	C/G	p.D110H	0.010	(NA)	0.020	0	10	58	0	0	68	Inf	1.65×10^−3^
rs35366573	Chr1:207958446	*CD46*	C/T	p.A290V	0.010	(NA)	0.020	1	8	59	0	1	67	10.5 (1.5–460.5)	1.02×10^−2^
rs6195	Chr5:142779317	*NR3C1*	T/C	p.N337S	0.010	(NA)	0.020	1	7	59	0	1	67	10.5 (1.5–460.5)	1.02×10^−2^
rs11670727	Chr19:50862768	*NAPSA*	C/T	p.A310T	0.020	(NA)	0.040	0	17	51	0	5	63	3.7 (1.2–13.1)	1.28×10^−2^
**Non-*RNF213* founder variant cases**															
rs16837497	Chr3:126180820	*ZXDC*	G/A	p.P562L	0.040	0.020	0.040	1	11	79	0	1	90	13.8 (2.0–593.3)	1.51×10^−3^
rs143744326	Chr1:152128212	*RPTN*	C/G	p.D110H	0.010	0.000	0.020	0	10	81	0	0	91	Inf	1.72×10^−3^
rs2290971	Chr7:148712092	*PDIA4*	G/A	p.T173M	0.020	0.040	0.010	0	10	81	0	1	90	Inf	1.72×10^−3^
rs2307145	Chr1:67833527	*IL12RB2*	G/C	p.Q426H	0.040	0.000	0.040	0	10	81	0	2	89	10.5 (1.5–458.2)	1.06×10^−2^
rs35366573	Chr1:207958446	*CD46*	C/T	p.A290V	0.010	0.000	0.020	1	8	82	0	1	90	10.5 (1.5–458.2)	1.06×10^−2^
rs6195	Chr5:142779317	*NR3C1*	T/C	p.N337S	0.010	0.000	0.020	1	8	82	0	1	91	10.5 (1.5–458.2)	1.06×10^−2^
rs11670727	Chr19:50862768	*NAPSA*	C/T	p.A310T	0.020	0.0017	0.040	0	17	74	0	5	86	3.6 (1.3–12.9)	1.38×10^−2^

Inf, infinite; NA, not applicable; A, ref allele; a, alt allele.

**Table 3:** Occurrence of the *RNF213* founder mutation among cases in multiple ethnic groups

Population	Ethnicity (Self-reported)	Total Samples	Founder Mutation
East Asian	Korean	11	8 (73%)
	Japanese	5	3 (60%)
	Chinese	5	1*^b^* (20%)
	Taiwanese	1	0
Pacific Islander	Hawaiian	4*^a^*	1*^c^* (25%)
	Filipino	5	0
Southeast Asian	Vietnamese	2	0
	Cambodian	1	0
	Laotian	1	0
	Malaysian	1	0
European	Caucasian	74	0
South Asian	Indian	2	0
	Sri Lankan	1	0
American	Native American	1	0
	Admixed American	6*^a^*	1*^c^* (17%)
African	African American	5	0

a Two of four Hawaiian and one of six Admixed American cases were determined to be of East Asian descent following genetic clustering.

b This individual was homozygous for the *RNF213* founder variant.

c These individuals were of East Asian descent based on genetic clustering (cluster with Korean and Japanese samples).

**Figure 2:**
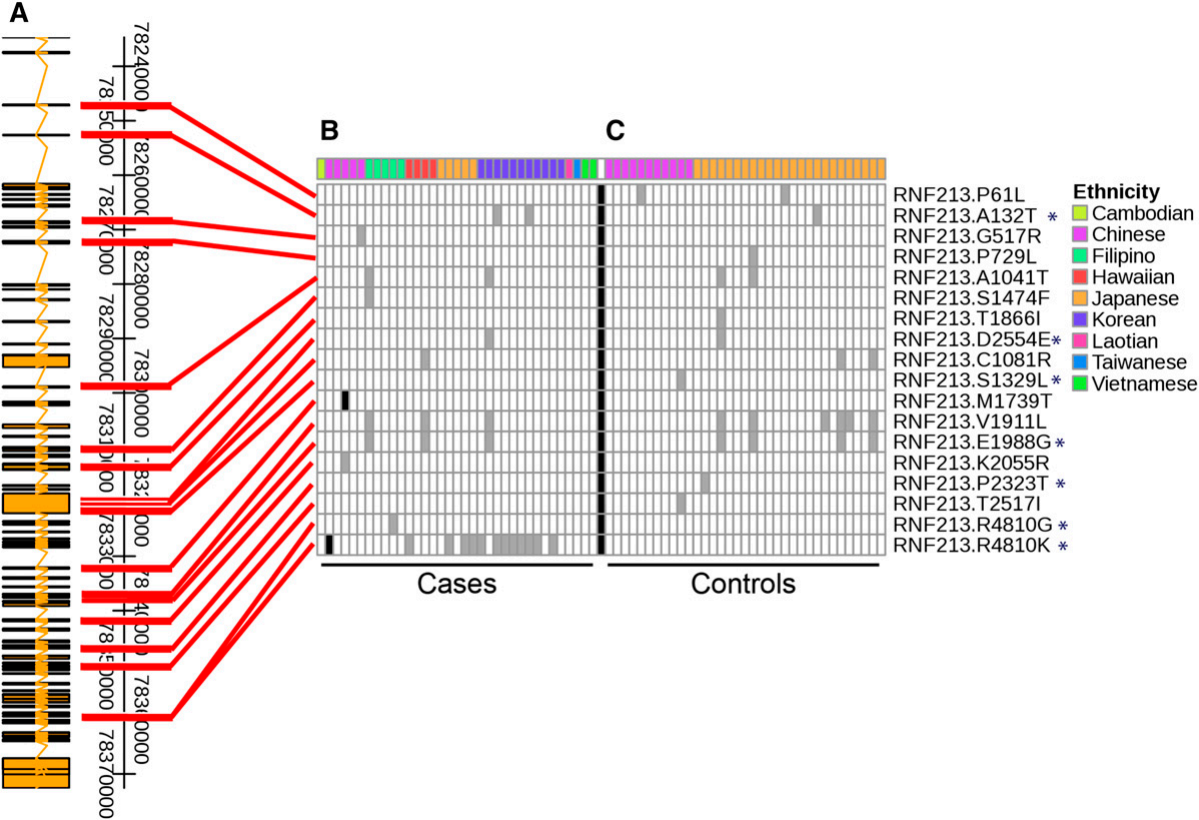
Comparison of nonsynonymous variants in *RNF213* between MMD cases and controls in the East Asian/Southeast Asian/Pacific Islander population. The schematic on the left shows the location of individual variants within the *RNF213* exons, with the founder mutation (p.R4810K) located in the bottom row (A). The MMD cases are grouped in (B) while the control cases are grouped in (C). The occurrence of individual variants (rows) within the *RNF213* gene across individual samples (columns) is indicated by a black (homozygous for alternative allele), gray (heterozygous), or white (no alternative allele) color scheme. Rows are ordered top (3′ end) to bottom (5′ end) by chromosomal position of the SNP and are labeled with the associated amino acid change. Ethnicities are shown per sample with distinguishing colors in the top row and associated legend. All identified variants within the *RNF213* gene are missense, including one (p.R4810G) that occurs in the same codon as the founder variant. *Previously published protein-coding *RNF213* variant (Kamada *et al.* 2011; Liu *et al.* 2011; Miyatake *et al.* 2012; Wu *et al.* 2012).

An additional 17 *RNF213* variants were identified in the Asian subgroup (Figure 2). Ten of these protein-coding variants were not previously reported as associated with MMD, and none was significantly enriched (*P* < 0.0001) in any ethnic group. A second variant was identified in the same amino acid codon as the *RNF213* founder mutation (p.R4810G) in one Filipino patient. Two homozygous variants were observed in Asian cases but not in any Asian controls (Figure 2B, shaded black squares). One of these samples was homozygous for the founder mutation p.R4810K, whereas the other was homozygous for the variant p.M1739T, a novel variant not present in dbSNP, not detected by 1000 Genomes (The 1000 Genomes Project Consortium 2012) or the NHLBI GO-ESP Exomes Project (Tennessen *et al.* 2012), nor previously identified in MMD.

### All protein-coding mutations in *RNF213* were missense mutations

We identified 142 synonymous and nonsynonymous *RNF213* variants in the protein coding sequence across 125 cases, and of the nonsynonymous variants only 15 had been previously identified (Kamada *et al.* 2011; Liu *et al.* 2011; Miyatake *et al.* 2012; Wu *et al.* 2012). Of these, 67 were rare *RNF213* variants (<5% population frequency) that cause a predicted coding change (Figure S2). A higher percentage (100%) of missense mutations was observed among all nonsynonymous *RNF213* variants than was observed among all other genes (*P* = 0.02). No frameshift, nonsense, or splice site mutations were observed. There are 26 additional previously published *RNF213* variants in MMD that we did not identify in this study, bringing the total number here and in the literature to 168 (Miyatake *et al.* 2012; Liu *et al.* 2011; Kamada *et al.* 2011; Wu *et al.* 2012). A full reference database of the 469 *RNF213* variants identified, including nonsynonymous, synonymous, and intronic, as well as the frequency for all MMD cases and controls can be found in Table S2.

We then determined whether non-*RNF213* founder mutation cases may be enriched for private, previously unobserved mutations in *RNF213* *vs.* control cases. We detected a total of 26 private mutations in *RNF213*, with 17 in MMD cases and 9 in controls (Figure S3). The difference is not statistically significant (*P* = 0.16), however, and could possibly be due to the relatively small number of samples tested.

### Additional gene variants associated with MMD in Asian cases

In addition to the *RNF213* founder mutation, the four most significantly enriched rare protein-coding variants identified in the East Asian, Southeast Asian, and Pacific Islander population included rs140134109 (*C19orf45*, p.M39V), rs146586179 (*ZNF783*, p.A267T), rs2241012 (*MLLT6*, p.A198T), and rs12718465 (*APOA1*, p.A61T) (Table 2). Each of these variants has been detected in the population before but in <5% of Asian individuals and in <1% of the 1000 Genomes Project. They are substantially enriched in the Asian MMD population, being present in 37% (*RNF213*, p.R4810K), 26% (*C19orf45*, p.M39V), and 20% (*ZNF783*, p.A267T; *MLLT6*, p.A198T; *APOA1*, p.A61T) of Asian MMD cases and absent/infrequent in controls.

### Unique gene variants in Caucasian MMD cases

The most highly enriched variant in Caucasian MMD was rs16837497 (*ZXDC*, p.P562L) (Table 2). This variant was present in 10 of 68 Caucasian cases (14.7%), with one homozygous and nine heterozygous cases (*P* = 7.93×10^−4^). However, this variant has a Caucasian population frequency of 4%, in contrast to the East Asian *RNF213* founder mutation, which has an East Asian population frequency of 0.2% according to the 1000 Genomes Project (The 1000 Genomes Project Consortium 2012). These data suggest that if the *ZXDC* p.P562L variant is causative for MMD, its penetrance in Caucasians is substantially lower than that of the *RNF213* founder mutation in East Asians. Additional nonsynonymous variants were enriched in the Caucasian MMD population but did not reach exome-wide significance (*P* < 10^−4^), and included rs143744326 (*RPTN*, p.D110H), rs35366573 (*CD46*, p.A290V), rs6195 (*NR3C1*, p.N337S), and rs11670727 (*NAPSA*, p.A310T) (Table 2).

### MMD-associated variants in multiethnic MMD cases lacking the *RNF213* founder mutation

Of the 91 MMD cases lacking the founder mutation, 22 were East Asian, Southeast Asian, or Pacific Islander and 67 were Caucasian (Table S1). We identified 1589 nonsynonymous variants associated with MMD, the seven most enriched of which are listed in Table 2. Because the majority of this dataset was Caucasian, some of the variants detected in the Caucasian ethnic study were replicated here, including *ZXDC* (p.P562L), *RPTN* (p.D110H), *CD46* (p.A290V), *NR3C1* (p.N337S), and *NAPSA* (p.A310T). We also observed enrichment of variants in *PDIA4* (rs2290971, p.T173M) and *IL12RB2* (rs2290971, p.Q426H).

To extend our analysis, we examined the presence of synonymous and intronic variants in the MMD cases lacking the *RNF213* founder mutation. Synonymous variants may play a role in disease mechanisms by exerting effects on protein splice variant expression, RNA secondary structure, and the rate of protein translation (Sauna and Kimchi-Sarfaty 2011). The novel variants identified here fell into three main functional groups: transcriptional regulation, immune system, and ECM/cytoskeleton. The six most enriched variants include rs3027849 (*HTATSF1*), rs139890952 (*ABCA7*), rs11666133 (*C3*), rs73120230 (*AMPH*), rs1437488 (*COBL*), and rs62242667 (*IQSEC1*) (Table S3). Interestingly, rs62242667 (*IQSEC1*) is located at 3p25.2 within a previously reported MMD susceptibility locus.

### Variant collapsing identifies additional MMD-associated genes

To identify genes significantly associated with MMD in the three case-control datasets, we used additional filtering to include only those variants that were protein-coding, had a low frequency (<5%) in the general population, were identified as single nucleotide variations (SNVs), and were enriched in MMD cases. These variants were then collapsed to genes enriched for variants for each dataset (see workflow in Figure S4). We identified 23 genes in the East Asian/Southeast Asian/Pacific Islander dataset, 24 genes in the Caucasian dataset, and 35 genes in the non-*RNF213* founder mutation dataset (Table S4). *OBSCN* (Gene ID: 84033) was ranked as the gene most enriched for variants (q = 5.31×10^−5^) in the Caucasian and the non-*RNF213* founder mutation cases with 53 variants in *OBSCN* unique to MMD cases, 35 unique to controls, and 19 present in both.

## Discussion

### Mutations in *RNF213* across an ethnically diverse MMD population

We have developed a summary of the mutations in *RNF213* in an ethnically diverse set of MMD cases, a library of which has been assembled to facilitate further exploration of this gene across ethnicities (Table S2). Previous work demonstrated that the *RNF213* p.R4810K founder mutation was most enriched in the Japanese population, present in the Korean and Chinese populations, and in individuals of Asian descent in the United States (Wu *et al.* 2012; Liu *et al.* 2011; Kamada *et al.* 2011; Cecchi *et al.* 2014). We independently confirmed this variant as the single most enriched mutation in Asian MMD cases and observed that this variant was not found in any of the self-declared Pacific Islander or Southeast Asian MMD cases but was present in 56% of the East Asian MMD cases. Moreover, the variant was not found in any of the Caucasian, South Asian, Admixed/Native American, or African American cases, offering further support of the previous discovery that the *RNF213* p.R4810K founder mutation originates in, and is specific to, the East Asian population.

### Insight into the *RNF213* protein

We identified other protein-coding variants in the *RNF213* gene, including many rare, nonsynonymous variants. We also observed a novel mutation at the same codon as the *RNF213* founder mutation, suggesting the possibility that other missense mutations at this arginine position may be associated with MMD. We also identified a novel homozygous variant p.M1739T that has not been observed in the 1000 Genomes or NHLBI GO-ESP projects and has no entry in dbSNP. Of particular note, all protein-coding variants in *RNF213* across all cases and controls were missense mutations. Numerous synonymous mutations were identified as well, but no frameshift, nonsense or splice site mutations were found, suggesting that severe loss of function mutations in *RNF213* may not be tolerated. Many variants, including the founder mutation, fall within a protein domain with no described function, whereas those variants occurring in the RING domain of RNF213 do not involve the conserved, and presumed essential, cysteine and histidine residues characterized in this family of ring finger proteins.

A significant barrier to the study of MMD is the lack of suitable models that recapitulate the disease. In the case of *RNF213*, mouse models show no cerebrovascular phenotype but some evidence for increased angiogenesis in a hindlimb ischemia model (Kobayashi *et al.* 2013; Ito *et al.* 2015), whereas a morpholino knockdown in zebrafish resulted in aberrant angiogenesis (Liu *et al.* 2011). There are myriad explanations for this, including that a complete absence of this protein may not reflect the potential gain-of-function of the p.R4810K variant. To examine the specific consequences of the *RNF213* p.R4810K founder mutation, Hitomi *et al.* (2013a) recently developed induced pluripotent stem cell (iPSC)-derived endothelial cell (EC) lines from three MMD patients (two of whom were homozygous for p.R4810K), one unaffected heterozygous subject, and two control subjects. Using this approach, they identified several genes with altered expression, including *Securin*, a gene involved in angiogenesis, chromosome stability, and DNA repair, and have further implicated *RNF213* p.R4801K protein in increasing genomic instability compared to wild-type (Hitomi *et al.* 2013b). However, it is still unclear how this relates to MMD and whether *RNF213* is indeed the causative gene in MMD.

### Identification of the novel *ZXDC* variant and involvement of the immune/autoimmune system

There is evidence for the association of autoimmune disorders, such as Graves disease and thyroid disease, with MMD, and some evidence that infection precludes MMD angiography (Bower *et al.* 2013; Houkin *et al.* 2012). We have also previously reported the expression of 165 significantly elevated autoantibodies in the sera of a multiethnic group of MMD patients compared with control subjects (Sigdel *et al.* 2013). The novel variant *ZXDC*, p.P562L was significantly associated with MMD in both Caucasian and non-*RNF213* founder mutation cases. This variant was found in 14.7% of Caucasian MMD cases, whereas the 1000 Genomes Project indicates it is present in 4% of Caucasian controls, representing a substantially lower enrichment than for the *RNF213* p.R4810K founder mutation in East Asians. These data suggest that if the *ZXDC* p.P562L mutation is causative for MMD, then it is a considerably lower penetrance mutation than the *RNF213* p.R4810K mutation in East Asians. The identification of the *ZXDC* p.P562L variant is intriguing, given its role in transcription of MHC Class II genes via interaction with *ZXDA* and *CIITA* (Al-Kandari *et al.* 2007). Patients with complete absence of MHC Class II gene expression exhibit severely compromised immune systems and frequently experience severe viral, bacterial, or fungal infections (Villard *et al.* 2001). The p.P562L amino acid change occurs in a region of the protein without any clearly defined functional domain, but which may have a subtle effect on the function of the activation domain of *ZXDC*, which is required to activate MHC Class II genes. The identification of other genes with known roles in the immune system, such as *APOA1*, *NR3C1*, *CD46*, *IL12RB2*, and *C3*, identified by the enriched variant and collapsing variant approaches, strongly suggests a role for an altered immune response in MMD. Future experiments involving a larger MMD patient population will further define the clinical diagnostic value of the *ZXDC* variant.

### Abnormal vascular smooth muscle cells and angiogenesis

Given that the affected cerebrovasculature in MMD is characterized by SMC proliferation, duplication/disruption of the elastic lamina, and aberrant angiogenesis, it is intriguing to speculate that mutations altering SMC contractibility, cell-to-cell contact, or the nature of the extracellular matrix (ECM) may shift SMCs from mature and nonproliferative to less differentiated and proliferative. There is an intimate relationship between vascular SMCs, ECs, and the ECM, aspects of which can modulate SMC phenotypes, as is the case in atherosclerosis (Orr *et al.* 2010). We identified *OBSCN*, a novel MMD-associated gene in Caucasian cases, and a number of other genes, such as *nebulin* and *titin*, through collapsing methodology. Obscurin is expressed in striated muscle and has essential roles in cell contractibility/adhesion, myofibrillogenesis, and cytoskeletal organization and, together with nebulin and titin, is necessary for the structure and contractibility of striated SMCs [as reviewed in Perry *et al.* (2013)]. Deletion of *OBSCN* in a zebrafish model resulted in defects in skeletal muscle, as well as in cardiac and neural development (Perry *et al.* 2013), whereas in disease, the p.R4344Q variant (not identified in this study) of *OBSCN* has been associated with hypertrophic cardiomyopathy, and other variants have been associated with various cancers (Perry *et al.* 2013). Other genes identified in this study have known roles in cytoskeletal organization, integrity, and remodeling, as well as in ECM deposition or remodeling, such as *RPTN*, *NAPSA*, *AMPH*, *COBL*, and *IQSEC1*. In the context of the abnormal SMC proliferation and angiogenesis observed in MMD, these variants may interfere with the complex and intricate communication between SMCs and EC required to maintain normally functioning vasculature.

### Toward potential Moyamoya disease mechanisms

In this study we identified variants in *RNF213* as well as other novel genes and gene variants unique to MMD patients, and although no additional founder mutations were discovered, we highlight the complexity of the underlying disease mechanisms. Our work provides additional support for the association of *RNF213* variants according to ethnicity, as previously suggested (Roder *et al.* 2011; Shimojima and Yamamoto 2009; Guo *et al.* 2009; Cecchi *et al.* 2014; Wu *et al.* 2012; Liu *et al.* 2011, 2012, 2013; Kamada *et al.* 2011). Several studies have further suggested ethnic differences in MMD phenotype and demographics, but a more thorough analysis is needed for this to be conclusive (Kraemer *et al.* 2008; Duan *et al.* 2012; Kuriyama *et al.* 2008; Starke *et al.* 2012). The limitations of these data set include the relatively small overall patient size, the mix of ethnicities present, and the requirement to use matched controls from a different data set. However, these limitations are largely due to the rarity of MMD in the population and the nature of the patient samples. By sequencing and analyzing the cases and controls internally and in parallel on a reasonably large cohort for such a rare disease, we were able to demonstrate support for a role of *RNF213* in MMD, as well as to identify new potential genes relevant to the disease. The genes containing the variants presented here currently have no known direct role in MMD but represent a rich set of candidates for future MMD research, including the development of disease models and potential advances in determining disease susceptibility and diagnosis. Future work will address the potential roles of these variants and genes in MMD across ethnicities.

## 
